# Mechanisms of Piezo1-Mediated Mechanotransduction in Thrombotic Diseases

**DOI:** 10.3390/ijms27188102

**Published:** 2026-09-11

**Authors:** Hanrong Dong, Yunlun Li, Wenqing Yang

**Affiliations:** 1 The First Clinical Medical College, Shandong University of Traditional Chinese Medicine, Jinan 250355, China; 16600641856@163.com (H.D.); li.yunlun@163.com (Y.L.); 2 Institute of Traditional Chinese Medicine Innovation, Shandong University of Traditional Chinese Medicine, Jinan 250355, China

**Keywords:** Piezo1, mechanical transduction, thrombosis, calcium signal, hemodynamics

## Abstract

The mechanical cation channel Piezo1 has been confirmed as a key membrane mechanical protein that can convert physical forces into biological signals, serving as a molecular hub linking hemodynamic stimulation with thrombotic diseases. Studying mechanical sensitive channels in the cardiovascular system is helpful for understanding the working mechanism of these channels and providing new therapeutic targets for thrombotic diseases. This article systematically summarizes the expression and functional roles of the Piezo1 channel in cell types involved in thrombosis, including platelets, endothelial cells, red blood cells, immune cells, etc. This article also discusses the mechanical gating structural basis of Piezo1 and its activation mechanism and downstream signaling pathways in the thrombosis-related mechanical microenvironment. Based on the existing evidence, Piezo1 has significant pathological significance in the occurrence and development of thrombotic diseases, and targeting Piezo1 may provide new strategies for anti-thrombotic treatment. Future research needs to further clarify the differential regulatory mechanisms of the Piezo1 channel in different hemodynamic environments and evaluate its safety and efficacy as a drug target.

## 1. Introduction

Thromboembolic disease is one of the leading causes of cardiovascular death globally, accounting for approximately one-quarter of all-cause mortality worldwide [1]. With the aging population, the burden of thromboembolic disease continues to escalate, resulting in complications such as myocardial infarction, stroke, and limb ischemia [2,3]. Thromboembolic disease encompasses venous thromboembolism (VTE) and arterial thromboembolic disease (ATE). ATE predominantly includes myocardial infarction (MI) and ischemic stroke, whereas VTE, which comprises deep vein thrombosis and pulmonary embolism, represents the third most common cardiovascular disease globally, with an annual incidence of approximately 0.1–0.2% in the general population [1,4]. VTE may lead to severe long-term sequelae, most notably chronic thromboembolic pulmonary hypertension. The economic burden is substantial, with VTE-related healthcare expenditure accounting for an estimated 0.05% to 0.18% of the gross domestic product across various countries in 2022 [5]. Acute venous and arterial thrombosis remain the leading causes of death in developed countries, with MI and cerebrovascular accident accounting for the highest proportion of thrombosis-associated fatalities [6]. Compounding this substantial burden, the fundamental therapeutic dilemma of current antithrombotic therapy lies in the inherent difficulty of inhibiting pathological thrombosis without compromising physiological hemostasis, as exemplified by the risk of gastrointestinal bleeding. Moreover, therapeutic options are particularly limited for patients at elevated bleeding risk, including those with cancer and the elderly, who face concurrent challenges of heightened thrombotic recurrence and bleeding susceptibility.

The current prevailing understanding of thrombus formation remains anchored in the classic Virchow’s triad—namely, abnormalities in blood flow, vascular wall dysfunction, and hypercoagulability—while also encompassing dysregulation of the coagulation-anticoagulation-fibrinolysis balance, interactions among multiple blood components, and homeostatic equilibrium between the arterial and venous endothelium [6,7]. In recent years, accumulating evidence has revealed that these prothrombotic determinants do not operate in isolation but are subject to continuous modulation by the local hemodynamic environment. Physiological shear stress signaling is essential for maintaining vascular homeostasis; however, under pathological conditions such as hypertension and arterial stenosis, local shear rates at diseased vascular sites are either increased or decreased. These aberrant mechanical stimuli can modulate the functional status of both vascular wall cells and circulating blood cells, thereby contributing to the initiation of thrombus formation [8,9]. In the arterial system, high shear stress activates platelets and promotes their aggregation, whereas in the venous system, low shear stress predisposes to blood stasis and facilitates the local enrichment of coagulation factors [10,11].

These mechanical stimuli are sensed by mechanosensitive (MS) channels on the cell surface, which respond to mechanical perturbations—including stretch, swelling, and fluid flow—within microseconds [12], thereby transducing physical signals into biochemical events such as ion influx. Among MS channels, Piezo1 is abundantly expressed in the cardiovascular system and critically involved in mechanotransduction processes including blood pressure regulation, vascular development [13], and hemodynamic force transmission in vascular cells. The activation of Piezo1 is governed by two principal models. The force-from-lipids model posits that fluid shear stress and extensional strain induce alterations in membrane tension and curvature, resulting in asymmetric pressure distribution across the inner and outer leaflets of the lipid bilayer and subsequent hydrophobic mismatch; these changes evoke conformational rearrangements arising from interactions between lipid acyl chains and the channel pocket, thereby activating the channel. This mechanism is consistent with the intrinsic three-dimensional architecture of Piezo1, which is evolutionarily tuned to sense membrane tension. In parallel, the force-from-filaments model proposes that, owing to the tight physical connections between Piezo1 and the cytoskeleton and/or extracellular matrix (ECM), mechanical movements of the cytoskeleton or ECM transmit pulling or pushing forces that gate the channel [14,15]. Thus, Piezo1 is capable of sensing global changes in membrane tension via the force-from-lipids model while simultaneously perceiving cytoskeletal tethering forces through the force-from-filaments model. These two mechanisms are complementary and together ensure that Piezo1 accurately interprets complex mechanical environments and elicits appropriate physiological responses.

Given the mechanosensitivity of Piezo1 and the complexity of its cellular effects, it is imperative to systematically delineate its prothrombotic or antithrombotic implications across different vascular beds and cellular contexts. Current research on this channel in the cardiovascular field has predominantly focused on hypertension, cardiac hypertrophy, heart failure, and atherosclerosis, whereas a systematic mechanistic synthesis in thrombotic diseases remains insufficient. This review summarizes the specific mechanisms by which Piezo1 participates in thrombus formation and the targeted therapeutic strategies available, with the aim of providing a reference for developing this channel as a therapeutic target for thrombotic disorders.

## 2. Structural Properties and Mechanosensing Mechanisms of the Piezo1 Channel

### 2.1. General Characteristics and Architecture of the Piezo1 Channel

The Piezo1 channel was first identified and named by Coste et al. in 2010 [16]. It is a non-selective cation channel permeable to Ca^2+^, Na^+^, and K^+^, and serves as a principal mechanosensory transducer in multiple mammalian tissues. The unique spatial conformation of Piezo1 constitutes the structural basis for its ability to sense, transmit, and transduce thrombosis-relevant mechanical signals [17]. Piezo1 adopts a homotrimeric architecture, with its three protein subunits assembling into a three-bladed, propeller-like structure [18], comprising highly curved peripheral blades and a central pore module. The opening of the central pore depends on mechanical signals transmitted from the peripheral blades, as the pore domain itself does not directly sense membrane stretch [14]; rather, the peripheral blades are responsible for detecting mechanical forces, including high shear stress, membrane tension, and compressive forces within the thrombus. Intracellular beams bridge the blades and the pore domain, relaying the mechanical signals perceived by the blades to the pore. Upon exposure to blood flow-derived shear stress, the peripheral blades undergo a flattening conformational change, followed by bending of the intracellular beams, which drives pore dilation and channel opening, thereby permitting substantial Ca^2+^ influx [19].

In the cardiovascular system, Piezo1 is expressed in vascular endothelial cells, vascular smooth muscle cells, platelets, and angiogenic smooth muscle cells [20]. It senses diverse mechanical stimuli, including fluid shear stress, membrane stretch, and mechanical traction, and opens within milliseconds to initiate downstream signaling cascades. Under physiological mechanical conditions, Piezo1 maintains vascular homeostasis. In contrast, under pathological mechanical microenvironments—such as high shear stress, low shear stress, or disturbed flow—aberrant Piezo1 activation promotes thrombus formation through driving platelet activation, endothelial barrier disruption, and neutrophil extracellular trap (NET) formation [18,21,22,23,24]. The specific mechanisms underlying these processes will be elaborated in subsequent sections.

### 2.2. Activation Patterns and Prothrombotic Effects of Piezo1 Channels in Distinct Hemodynamic Environments

Aberrant blood flow serves as the direct trigger for pathological Piezo1 channel activation during thrombogenesis. At sites predisposed to thrombosis—such as arterial stenoses, venous stasis, and flow disturbances—alterations in flow velocity, vascular geometry, and blood viscosity give rise to abnormal shear stress profiles. The hemodynamic characteristics vary across different vascular beds, which in turn determine the differential activation patterns of Piezo1. In the arterial system, lesions such as stenosis cause marked elevations in local blood flow velocity, generating high shear stress (HSS). In contrast, the venous system, characterized by slow blood flow and post-valvular stasis, tends to develop low shear stress (LSS) conditions. At vascular curvatures or bifurcations, oscillatory shear stress (OSS) may arise [25]. These aberrant mechanical forces act directly on endothelial cells of the vascular wall as well as circulating platelets, erythrocytes, and immune cells, modulating Piezo1 channels on their surfaces and initiating the pathological process of thrombosis [26]. In the following sections, we will dissect the specific roles of Piezo1 in thrombus formation from the perspectives of distinct shear stress regimens, different cell types, and various vascular beds.

## 3. Differential Activation and Prothrombotic Mechanisms of Piezo1 Under Distinct Hemodynamic Shear Stress Regimens

### 3.1. Low Shear Stress: Piezo1-Mediated Endothelial Barrier Disruption Driving Thrombosis

The prothrombotic pathways activated by Piezo1 differ depending on the magnitude of shear stress. Under low shear stress conditions, such as those occurring at the site of carotid artery ligation, Piezo1 signaling promotes nuclear translocation of Yes-associated protein (YAP) and subsequent upregulation of vascular cell adhesion molecule-1 (VCAM-1) in endothelial cells. Elevated VCAM-1 expression creates a pro-inflammatory microenvironment through leukocyte recruitment, which indirectly facilitates platelet adhesion and activation [27]. Concurrently, Piezo1 signaling interacts with PECAM-1, and this crosstalk also contributes to platelet–endothelial adhesion [27]. Additional studies have demonstrated that prolonged exposure to low shear stress (0.1 dyne/cm^2^) under elevated hydrostatic pressure mediates aberrant opening of Piezo1 channels and significantly reduces the expression of VE-cadherin, an adherens junction protein critical for maintaining endothelial barrier integrity, at cell–cell junctions. This compromise of endothelial barrier function leads to subendothelial exposure of tissue factor, thereby triggering the extrinsic coagulation cascade, and concurrently increases vascular permeability, facilitating plasma extravasation and inflammatory cell infiltration—all of which represent initial events in thrombogenesis. These effects can be reversed by Piezo1 inhibition [24,27,28].

### 3.2. High Shear Stress: Piezo1-Mediated Platelet Activation Driving Thrombosis

In high-shear regions such as arterial stenoses, platelets are capable of directly sensing acute hemodynamic changes and exhibit a more rapid response to supraphysiological levels of extensional strain. This high shear stress, mediated by platelet Piezo1 channels, induces substantial extracellular Ca^2+^ influx, which in turn promotes the release of von Willebrand factor (VWF) from α-granules. Released VWF binds to platelet glycoprotein Ibα (GPIbα) and mediates the interaction between platelet integrin αIIbβ3 and fibrinogen, ultimately culminating in platelet aggregation and arterial thrombus formation [28,29]. In vivo experiments have shown that Piezo1 inhibition significantly prolongs vascular occlusion time in mouse models of high shear-induced arterial thrombosis, further substantiating the central role of Piezo1 in high shear stress-mediated arterial thrombogenesis [28].

### 3.3. Oscillatory Shear Stress: Piezo1-Driven Endothelial Inflammation and Barrier Injury in Thrombosis

OSS, a flow pattern predominantly occurring at vascular curvatures and bifurcations, can directly induce the phenotypic conversion of vascular endothelial cells (ECs) from a normal anticoagulant state to a pro-inflammatory and procoagulant phenotype. OSS activates Piezo1 in endothelial cells, leading to Ca^2+^ influx and subsequent activation of the Ca^2+^/CaM/CaMKII signaling axis, along with downstream focal adhesion kinase (FAK) and Src kinases. These signaling events promote YAP dephosphorylation at serine residue 127, ultimately eliciting endothelial inflammatory responses that accelerate thrombus progression. Beyond the aforementioned pathways, OSS also triggers excessive production of reactive oxygen species (ROS), intracellular Ca^2+^ overload, phosphorylation of VE-cadherin, and upregulation of adhesion molecules including ICAM-1 through Piezo1 activation, culminating in endothelial barrier disruption [30] and a pro-inflammatory phenotypic switch, which constitutes an early critical event in thrombogenesis. The breakdown of endothelial barrier integrity translates into enhanced vascular permeability, permitting procoagulant substances and inflammatory mediators to more readily penetrate the endothelial lining and expose subendothelial tissue factor (TF), as evidenced by marked increases in TF mRNA and protein expression [25]. This not only promotes vascular remodeling but also provides a structural foundation for the initiation of the coagulation cascade. Thus, OSS activates the endothelial Piezo1, induces endothelial inflammatory responses and disrupts the vascular barrier, which constitutes an important link in the transformation from hemodynamic abnormalities to the pre-thrombotic state [30].

## 4. Platelet Piezo1: A Core Effector in Arterial Thrombosis

Transcriptomic and proteomic evidence has confirmed Piezo1 expression in platelets [23,29] and arterial thrombosis is critically dependent on the mechanoresponsiveness of the platelet Piezo1 pathway [23]. This derives from two major characteristics of platelets. First, platelets are directly exposed to the mechanical environment of blood flow and can perceive changes in extensional strain without intermediation by vascular wall structures [29]. Second, as anucleate cells, platelets bypass transcriptional, translational, and protein synthesis processes, enabling them to complete the entire sequence from mechanoperception to functional output within milliseconds to seconds. Under pathological high-shear conditions such as arterial stenosis, Piezo1 channels are directly gated, promoting platelet hyperactivation [23,29].

The platelet Piezo1 channel transduces changes in membrane tension into calcium influx—an indispensable signaling event for platelet activation. Under physiological conditions, resting platelets adapt to low-level shear stress, thereby maintaining their quiescent state and hemostatic balance. However, under pathological conditions such as vascular stenosis and vasospasm, aberrantly elevated shear stress overactivates platelet Piezo1, triggering shear-induced platelet aggregation (SIPA), which constitutes a critical pathological basis for major cardiovascular events including myocardial infarction and ischemic stroke. SIPA thrombi are composed primarily of platelets and VWF, with minimal fibrin content; under high-shear flow, these components self-assemble into a cable-like structure spanning the vascular lumen, effectively resisting blood flow impact [31]. Upon activation, Piezo1 channels trigger Ca^2+^ influx and participate deeply in thrombus formation through modulation of downstream signaling networks and energy metabolism. In this chapter, we systematically dissect the downstream signaling network of the Piezo1 channel and its mitochondrial pathways, with the aim of elucidating its central role in thrombogenesis.

### 4.1. The Piezo1-Mediated Mechanical Signal Transduction Network: From Mechanical Sensing to Pro-Thrombotic Effects

Accelerated blood flow exerts various mechanical forces on the vasculature, including wall strain rate, fluid shear stress, and extensional strain, among others [24]. Acute changes in extensional strain at the arterial level are mechanically sensed by platelets [24]. leading to activation of platelet Piezo1 channels and robust calcium signaling [32], which in turn induces deformation of the platelet plasma membrane and cytoskeleton [24], as well as platelet activation, aggregation, and arterial thrombus formation [18,21,22].

Piezo1-mediated Ca^2+^ influx serves as the central driving force for platelet activation, subsequently initiating a cascade of signal amplification and synergistic events. First, the Ca^2+^ signal is relayed via Pannexin1 channel-mediated ATP release to P2X1 channels, which amplify the Ca^2+^ signal by 2- to 3-fold [22], forming an “extensional strain sensing mechanosome” cooperatively driven by Piezo1 (primary strain sensor) and P2X1 (secondary signal amplifier) [24]. In addition to calcium signaling, Pannexin1 concurrently amplifies GPVI procoagulant signals and transmits them to platelet P2X1 receptors [25]. The synergistic action of GPVI signaling and amplified calcium signals promotes platelet procoagulant activity and facilitates local fibrin formation, contributing to inflammatory hemostasis [27]. Second, Ca^2+^ influx enhances the release of ADP and ATP from dense granules, with ADP and ATP signals delivered to platelet P2Y1 and P2X1 receptors, respectively, further reinforcing calcium signaling through positive feedback. Furthermore, the incoming calcium signal activates calpain, which cleaves cytoskeletal proteins including talin and actin, as well as STIM1 oligomers, promoting myosin light chain phosphorylation. The intracellular traction forces generated by this phosphorylation can induce spatially restricted, Piezo1-mediated local calcium transients in the absence of external force [28]. Collectively, these processes drive platelet morphological transformation from a discoid to a pseudopod-bearing activated state, accompanied by cytoskeletal reorganization [29]; and indirectly modulate platelet surface ORAI1 channel activity while triggering conformational activation of integrin αIIbβ3, thereby lowering the platelet activation threshold. Finally, Ca^2+^ influx facilitates TMEM16F-mediated phosphatidylserine exposure and activates the PI3K/Akt signaling pathway. TMEM16F-induced platelet swelling and PI3K activation reciprocally enhance Piezo1 activity [24], with PI3K promoting the phenotypic switch of platelets toward a procoagulant state [25].

The aforementioned process is independent of classical activation pathways driven by soluble agonists such as ADP and thrombin, and does not rely on intraplatelet chemical amplification loops [24,33]. Although this mechanotransduction pathway converges with the classical mode at the level of downstream effector responses—including integrin activation and granule release—its upstream triggering mechanism is fundamentally distinct and is characterized by markedly faster response kinetics [25].

### 4.2. Mitochondrial Pathway: Ca^2+^ Signaling Energizes Platelet Activation and Regulates Apoptosis

In addition to initiating platelet activation signals, Piezo1-mediated Ca^2+^ signaling regulates platelet procoagulant function and cell fate through mitochondrial pathways.

**Figure 1:**
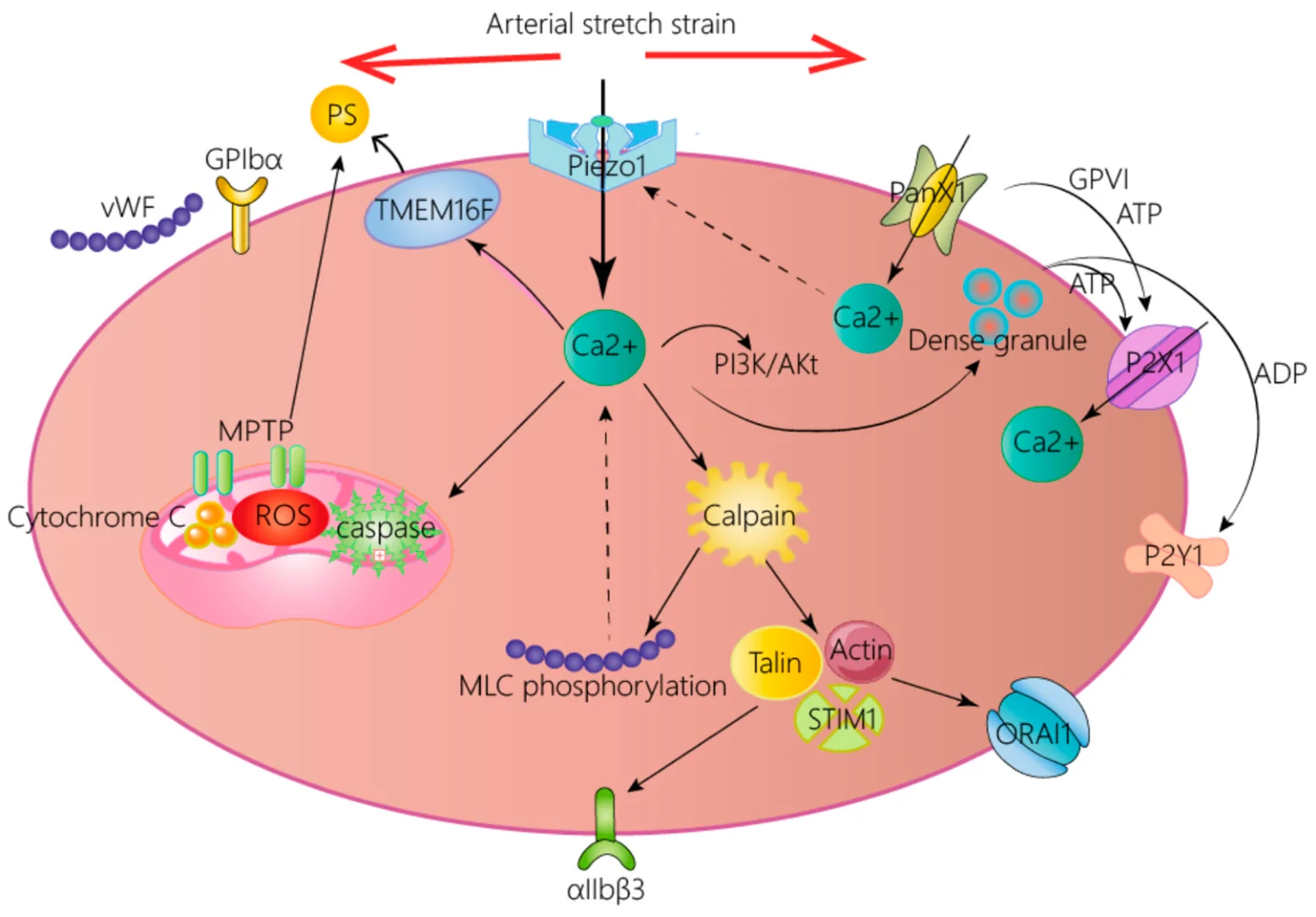
The mechanism of platelet piezo1 channels in the process of thrombosis. Stretching at the arterial level activates the Piezo1 channel, triggering the initial Ca^2+^ influx, promoting VWF production, and the binding of VWF and GPIbα receptors. The Ca^2+^ signal is transmitted to the P2X1 channel via the ATP release mediated by Panx1 channel, amplifying the Ca^2+^ signal by 2–3 times and amplifying the procoagulant signal of GPVI. GPVI is presented to P2X1. The Ca^2+^ signal enhances the release of dense granules to ADP/ATP, which is presented to the P2Y1/P2X1 channels, and the positive feedback strengthens the calcium signal. The Ca^2+^ signal activates calpain to cleave talin/Actin cytoskeleton and STIM1, promoting the phosphorylation of MLC, driving cytoskeleton remodeling and pseudopod formation; the intracellular traction generated after phosphorylation induces Piezo1-mediated calcium transient and indirectly regulates ORAI1 and activates αIIbβ3. The Ca^2+^ signal causes TMEM16F to mediate the exposure of PS, activating the PI3K/Akt signaling pathway, and the PI3K reversely enhances the activity of Piezo1. The Ca^2+^ signal is taken up by mitochondrial MCU, and Ca^2+^ overload induces MPTP opening, promoting ROS burst, mitochondrial redox imbalance, cytochrome c release, and caspase cascade activation, initiating apoptosis and amplifying PS exposure. The solid and dashed arrows have no substantive difference.

Platelet mitochondria serve as the metabolic hub [30]. Under resting conditions, platelet mitochondria maintain basal metabolic activity, and moderate Ca^2+^ signaling provides energy for platelet activation. However, under hypertensive conditions, aberrant hemodynamic signals mediate Piezo1 channel opening, and the elevated cytosolic Ca^2+^ is taken up by mitochondria, which activates the tricarboxylic acid (TCA) cycle and promotes ATP synthesis, thereby providing energy for platelet shape change, granule release, and integrin activation. Specifically, this manifests as opening of the mitochondrial permeability transition pore (mPTP), mitochondrial redox imbalance, and ROS burst, accompanied by increased cytochrome c release and activation of the caspase cascade, ultimately inducing phosphatidylserine (PS) exposure and platelet apoptosis [23]. These processes not only compromise platelet function but also exacerbate the vascular microenvironment through the release of pro-inflammatory and pro-apoptotic mediators, establishing a prothrombotic vicious cycle [23]. Of note, although the above-mentioned molecular pathways of mitochondrial Ca^2+^ uptake and ATP generation have been validated in platelets, direct evidence that Piezo1 triggers MCU-dependent mitochondrial Ca^2+^ uptake remains insufficient at present. The above mechanisms by which platelets promote thrombosis through Piezo1 are schematically illustrated in Figure 1.

## 5. Endothelial Piezo1: From Vascular Homeostasis to Prothrombotic Microenvironment

Having detailed the mechanisms by which platelet Piezo1 drives arterial thrombus formation in the preceding sections, we now turn to the endothelium. Vascular endothelial cells are continuously exposed to blood flow-derived shear stress, a critical signal that regulates endothelial function, vascular homeostasis, and thrombus formation. Shear stress is initially sensed by the glycocalyx, a polysaccharide–protein complex covering the endothelial surface. Acting as an apical membrane flow sensor, the glycocalyx amplifies and transduces shear signals into local increases in membrane tension [34], which are accompanied by stretching deformation of the lipid bilayer. These processes collectively drive Piezo1 channel opening, establishing Piezo1 as a key molecular link between macroscopic hemodynamic derangements and microcellular signal transduction [34,35].

Different vascular regions experience distinct flow patterns. In straight segments of the vasculature, blood flow is unidirectional and uniform in velocity, generating predominantly steady laminar shear stress. By contrast, at vascular curvatures, bifurcations, and stenotic sites, the flow direction is variable and velocity uneven, giving rise to disturbed flow characterized mainly by OSS. Disturbed flow aberrantly activates Piezo1 channels, driving endothelial inflammatory responses, barrier disruption, and the establishment of a procoagulant phenotype. As a principal fluid shear sensor on endothelial cells [36], Piezo1 mediates antithrombotic protection under physiological laminar flow, whereas in pathological disturbed flow microenvironments—such as those associated with inflammation and hypertension—it promotes prothrombotic transformation. In this chapter, we will elaborate on the dual regulatory roles of Piezo1 in endothelium under different flow patterns, as well as the prothrombotic mechanisms induced by inflammation and hypertension, and finally discuss potential therapeutic strategies targeting endothelial Piezo1.

### 5.1. Protective Role of Endothelial Piezo1 Under Laminar Flow Conditions

**Figure 2:**
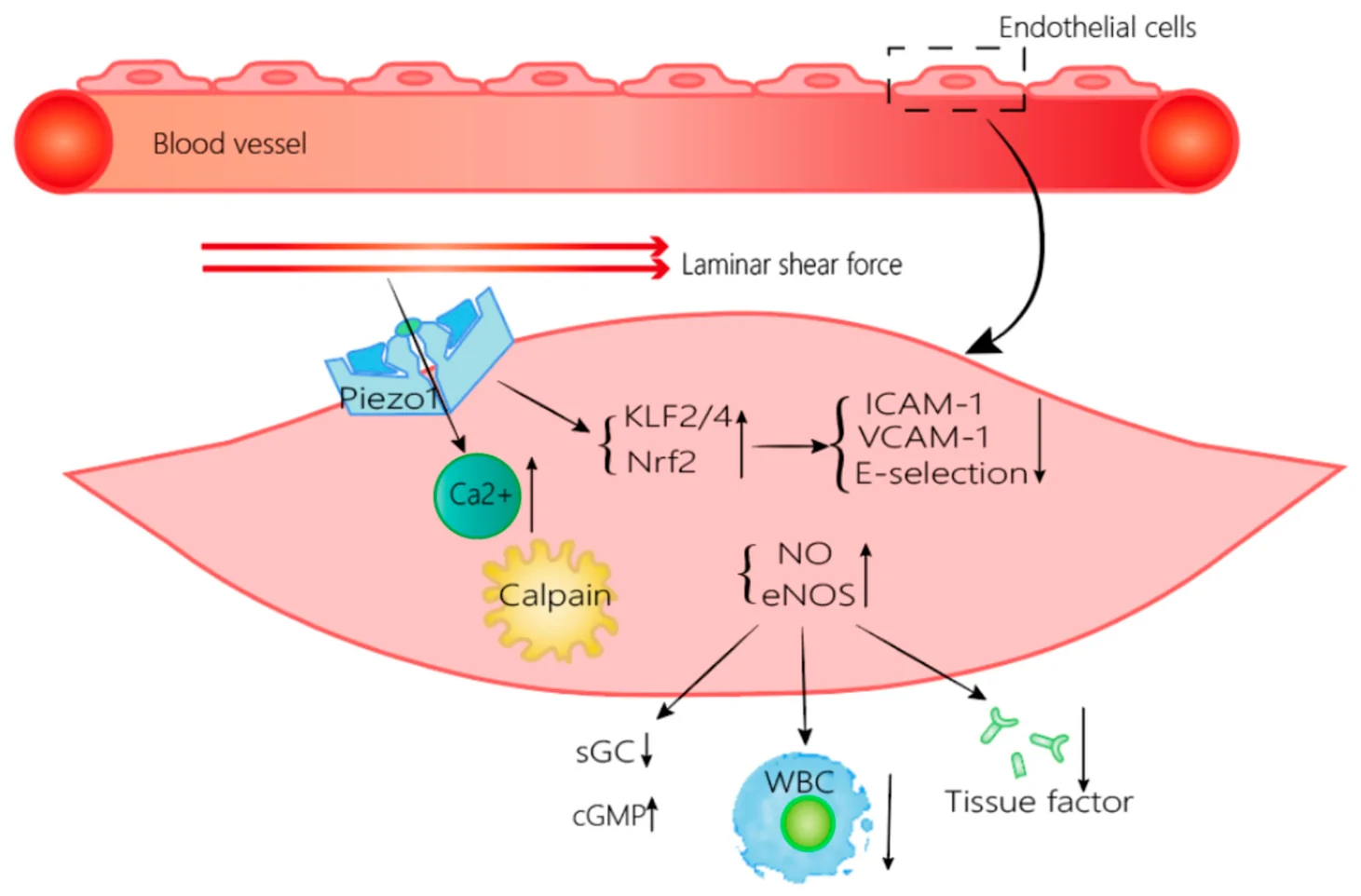
Laminar shear stress regulates the Piezo1 channel in endothelial cells. The activated Piezo1 remodels the actin cytoskeleton through the Ca^2+^/calpain pathway; it promotes eNOS phosphorylation and increases NO production. NO downregulates sGC and increases cGMP to inhibit platelet activation and aggregation, relaxes vascular smooth muscle, inhibits leukocyte adhesion and the synthesis of coagulation factors such as tissue factor, and downregulates adhesion molecules. The activated Piezo1 upregulates anti-inflammatory or antioxidant transcription factors such as KLF2, KLF4, and Nrf2, and inhibits the expression of pro-inflammatory/pro-adhesive factors such as ICAM-1, VCAM-1, and E-selectin to prevent thrombosis.

Under homeostatic physiological conditions, laminar shear stress maintains endothelial cells in a spindle-shaped morphology aligned with the direction of flow, preserves barrier integrity and an anticoagulant phenotype, and promotes the production of glycosylated products and anticoagulant substances, thereby sustaining hemodynamic stability and exerting antithrombotic effects [37]. Laminar shear stress activates Piezo1 channels on the endothelial surface, eliciting moderate Ca^2+^ influx. Upon activation, Piezo1 promotes actin cytoskeletal remodeling through the Ca^2+^/calpain pathway, aligning endothelial cells in the flow direction and minimizing flow disturbance; concurrently, it facilitates eNOS phosphorylation and activation, augmenting nitric oxide (NO) production and release. As a critical vasodilator, NO exerts pleiotropic antithrombotic actions through multiple pathways, including inhibition of platelet activation and aggregation via activation of soluble guanylate cyclase and elevation of cyclic guanosine monophosphate levels, relaxation of vascular smooth muscle, suppression of leukocyte adhesion, and inhibition of tissue factor and other coagulation factor synthesis, as well as downregulation of adhesion molecule expression [38]. Furthermore, under physiological laminar shear stress, Piezo1 activation upregulates the expression of anti-inflammatory and antioxidant transcription factors, including KLF2, KLF4, and Nrf2, which function predominantly to suppress pro-inflammatory and proadhesive factors such as ICAM-1, VCAM-1, and E-selectin, thereby reinforcing the antithrombotic phenotype [39]. The detailed mechanisms are illustrated in Figure 2.

At sites of vascular curvature, bifurcation, and stenosis, blood flow exhibits disturbed flow characterized by multidirectional, non-uniform velocity and frequently associated with vortices. This alteration in flow pattern generates local low-amplitude oscillatory shear stress, which can overactivate endothelial cells and platelets, promote the accumulation of thrombin and thromboxane A_2_, and, through opening of Piezo1 channels [39], trigger endothelial barrier disruption, release of inflammatory cytokines, and establishment of a procoagulant phenotype, ultimately culminating in a prothrombotic microenvironment.

### 5.2. Prothrombotic Transformation of Endothelial Piezo1 Under Disturbed Flow and Hypertension

At vascular curvatures, bifurcations, and stenotic segments, OSS hyperactivates platelets, promoting the accumulation of thrombin and thromboxane A_2_, while concurrently activating Piezo1 channels on endothelial cells [19] and the nuclear factor κB (NF-κB) inflammatory pathway in endothelial cells, OSS triggers endothelial barrier disruption, upregulates adhesion molecules and inflammatory cytokines, and enhances Gq/11–integrin signaling, collectively inducing an endothelial phenotypic switch from an anticoagulant to a procoagulant state. These processes culminate in endothelial inflammation and barrier compromise, ultimately establishing a prothrombotic microenvironment [39].

This Piezo1-driven inflammatory response further promotes leukocyte recruitment and activation. Elevated leukocytes induce clustering of the adhesion molecule ICAM-1, and the mechanical forces generated during this process can in turn activate Piezo1, triggering intracellular Ca^2+^ elevation and downstream phosphorylation events involving Src and proline-rich tyrosine kinase 2 (PYK2), ultimately leading to endothelial barrier opening. Sustained inflammation and hyperpermeable endothelium expose subendothelial tissue factor and promote platelet adhesion and aggregation, which likewise constitute critical steps in thrombogenesis [33].

**Figure 3:**
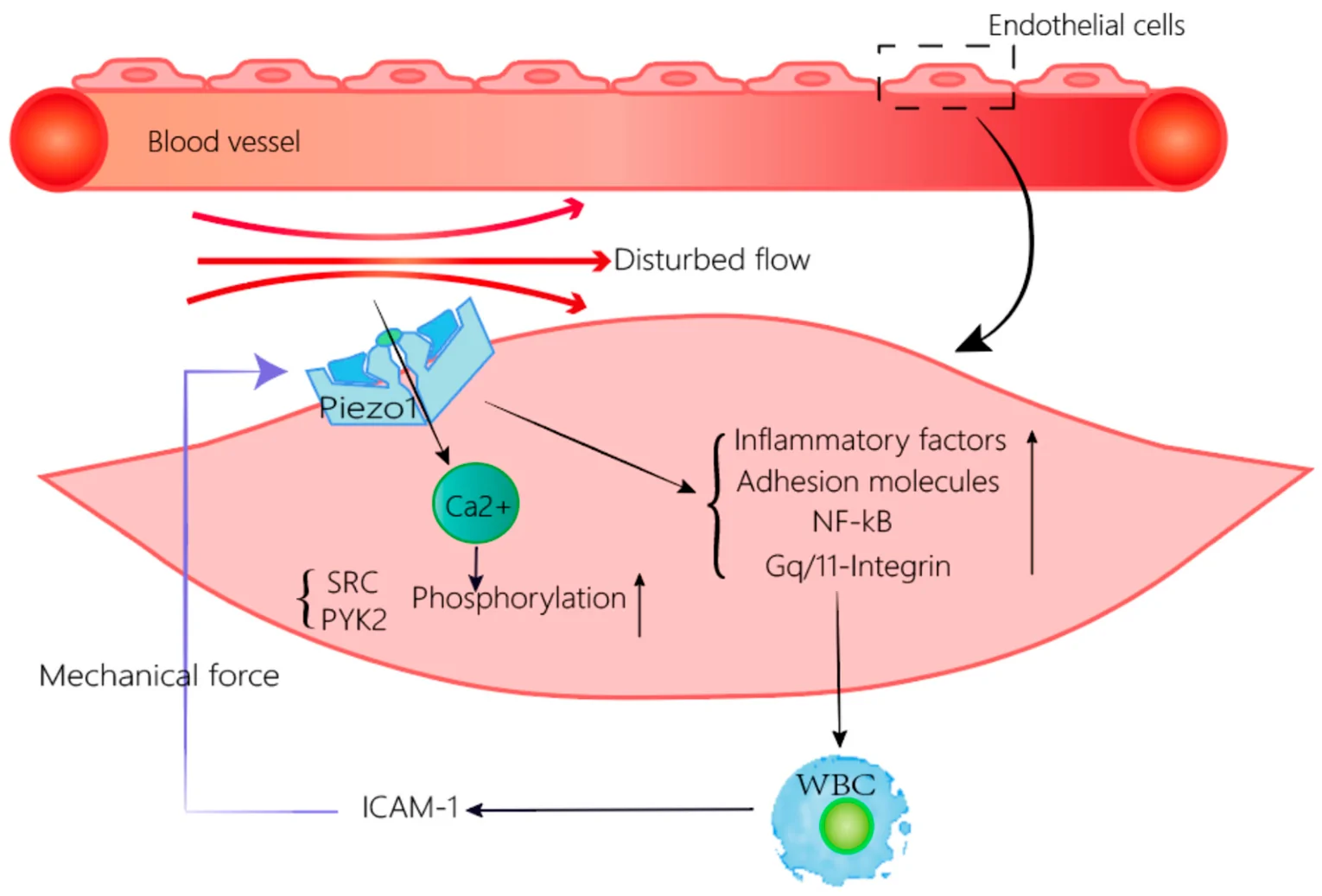
Disturbed flow shear stress regulates the Piezo1 channels of endothelial cells. Oscillatory shear stress activates Piezo1 in endothelial cells, leading to a substantial Ca^2+^ influx, concurrent activation of the NF-κB pathway, upregulation of adhesion molecules and inflammatory cytokines, enhanced Gq/11-integrin signaling, and increased endothelial permeability. Leukocyte activation is promoted, which in turn induces ICAM-1 clustering. The mechanical forces generated during this process activate Piezo1, resulting in elevated intracellular Ca^2+^ levels and subsequent phosphorylation of downstream SRC and PYK2. In hypertension, abnormal hemodynamic forces activate endothelial Piezo1 channels, triggering the release of VWF, PAF and TXA_2_. The ensuing platelet activation consumes NO and inhibits endothelial eNOS, thereby further exacerbating endothelial dysfunction and the inflammatory response.

In addition to the inflammatory milieu, hypertensive conditions are characterized by aberrant hemodynamic forces that also activate endothelial Piezo1 channels, inducing the release of prothrombotic factors including von Willebrand factor, platelet-activating factor, and thromboxane A_2_, which promote platelet activation. Activated platelets, in turn, consume endothelial nitric oxide and suppress eNOS expression, further exacerbating endothelial dysfunction. The activated platelets, in turn, will further intensify the inflammatory response of the endothelial cells, forming a vicious cycle that promotes thrombosis [23]. The aforementioned mechanisms are illustrated in Figure 3.

Nevertheless, the aforementioned pathological effects of Piezo1 are not irreversible. Endothelial-specific Piezo1 knockout has been shown to significantly reduce inflammatory leukocyte extravasation and block downstream pro-inflammatory and prothrombotic signaling, thereby indirectly retarding thrombus formation [33]. However, under conditions where inflammatory stimuli have already abolished basal Ca^2+^ oscillations and established a procoagulant phenotype, Piezo1-mediated physiological Ca^2+^ signaling may become impaired or desensitized. Under these circumstances, pharmacological activation of Piezo1 with its specific agonist Yoda1 can restore basal Ca^2+^ oscillations and attenuate inflammatory responses. Piezo1 activation upregulates the flow-sensitive transcription factors KLF2 and KLF4, which in turn suppress NF-κB phosphorylation and intercellular adhesion molecule-1 (ICAM-1) expression, while concurrently reducing tissue factor levels, diminishing procoagulant activity, and enhancing endogenous anticoagulant capacity. Concomitantly, thrombin and factor Xa generation, as well as fibrin deposition in vivo, are significantly reduced, collectively exerting anti-inflammatory and antithrombotic effects both in vitro and in vivo [40]. Collectively, these findings indicate that pharmacological opening of Piezo1 can reverse the procoagulant phenotype of endothelial cells under specific inflammatory contexts, thereby conferring protective effects. This is not contradictory to its prothrombotic role under high shear stress conditions, but rather exemplifies the dual regulatory functions of Piezo1 in distinct microenvironments.

Based on the aforementioned bidirectional regulatory mechanisms of Piezo1 in endothelial cells, Piezo1 holds considerable promise as a potential therapeutic target for modulating thrombus formation through maintenance of hemodynamic equilibrium. However, simple inhibition or opening of Piezo1 may not represent a universally applicable intervention strategy; rather, tailored approaches based on local flow patterns are warranted. In vascular regions dominated by laminar flow, preserving basal Piezo1 activity may contribute to endothelial homeostasis, whereas in areas exposed to disturbed flow, moderate inhibition of aberrant Piezo1 overactivation may block prothrombotic transformation. In the late stage of inflammation, Piezo1 agonists may be applicable for the treatment of established inflammatory thrombotic disorders, rather than being limited to prophylactic use. Furthermore, physical interventions such as stent design to improve local flow patterns, in combination with Piezo1-targeted pharmacotherapy, may offer novel approaches to rectify aberrant mechanical signal transduction and restore the antithrombotic phenotype of the vasculature.

## 6. Erythrocytic Piezo1: Thrombus Stabilization and Contraction

Erythrocytes participate in thrombus architecture and stabilization through the Piezo1 channel. The mechanical behavior of erythrocytes and its correlation with Piezo1 activation are particularly relevant during the late stages of clot contraction and in microcirculatory dysfunction. Under physiological conditions, Piezo1 mediates moderate erythrocyte dehydration to maintain deformability. However, under pathological mechanical conditions such as clot contraction and blood stasis, excessive Piezo1 opening in erythrocytes triggers calcium influx and cellular dehydration, thereby enhancing thrombus density and stability. Dysregulated Piezo1 activity on sickle erythrocytes also markedly increases thrombotic risk. This chapter will address the regulatory mechanisms of Piezo1 in normal erythrocytes during clot contraction, as well as the prothrombotic phenotype resulting from Piezo1 dysregulation in sickle erythrocytes, thereby elucidating the pathophysiological significance of erythrocytic Piezo1 in thrombosis and microvascular obstruction.

### 6.1. Erythrocytic Piezo1 and Thrombus Contraction

Piezo1 is one of the core mechanosensory proteins in mature erythrocytes. Under physiological conditions, Piezo1 mediates moderate erythrocyte dehydration to maintain deformability as red blood cells traverse the narrow splenic sinusoids. However, when erythrocytes are subjected to compression, such as during thrombus contraction, Piezo1 in compressed red blood cells is activated, triggering Ca^2+^ influx and phosphatidylserine (PS) exposure. This subsequently opens the downstream Gárdos channel, leading to K^+^ efflux and cellular dehydration, which reduces erythrocyte volume and compromises deformability, rendering the cells more prone to rupture. Ultimately, this process enhances thrombus density and stability.

As erythrocyte dehydration and clot contraction proceed, erythrocytes located in the core of the thrombus, subjected to mechanical compression by activated platelets, gradually transform from the normal biconcave disc shape into convex, irregular polyhedral structures, which Tutwiler and colleagues designated as “polyhedrocytes” [41]. The contractile forces generated by activated platelets retract the fibrin network [42], such that polyhedrocytes become encased within a meshwork of fibrin and platelets, with tightly compressed erythrocytes in the interior [41]. The interstices between polyhedrocytes are minimal, conferring a nearly impermeable seal [41,43], that effectively seals wound bleeding under physiological conditions [42], while pathologically resisting plasminogen penetration and thereby contributing to thrombolytic resistance. This structure has been demonstrated in both arterial and venous thrombi in humans and mice [42], representing a physical defense barrier that reduces thrombus permeability, encases the clot, and resists fibrinolytic degradation. Mechanical compression-induced deformation of erythrocytes serves as a prerequisite for Piezo1 to sense mechanical signals. Upon activation, Piezo1 channels promote phosphatidylserine (PS) exposure and thrombin generation, which in turn further enhance platelet contractile force and amplify thrombus retraction.

### 6.2. Aberrant Piezo1 Activation in Sickle Erythrocytes

Sickle erythrocytes exhibit markedly increased rigidity [42], and hypoxia-induced sickling alters erythrocyte morphology and membrane tension. This intrinsic mechanical force activates Piezo1 on the surface of sickle erythrocytes, leading to increased Piezo1 channel density and open probability, with enhanced sensitivity to mechanical stress, followed by elevated intracellular calcium and activation of downstream TMEM16F. Subsequently, the potent procoagulant phospholipid PS is exposed on the erythrocyte surface [37], and PS^+^ microparticles are released, further expanding the procoagulant surface area and increasing thrombotic risk. Exposed PS provides a binding platform for coagulation factors such as FXa and FVa within the prothrombinase complex, facilitating local thrombin generation. Thrombin, in turn, activates platelets and enhances their contractility, while also activating platelets and endothelial cells to release proadhesive mediators that augment sickle erythrocyte–endothelial adhesion. Collectively, these effects establish a prothrombotic biochemical milieu. Moreover, sickle erythrocytes exhibit enhanced thrombin-generating capacity under hypoxic conditions, which synergizes with the Piezo1–TMEM16F activation pathway to promote thrombosis [44].

## 7. Piezo1 in Immunothrombosis: Mechanisms of NETs

Piezo1 mediates immunothrombosis. Elevated shear stress causes endothelial injury and, through opening of Piezo1 and store-operated calcium channels such as ORAI1, increases intracellular calcium concentrations, thereby establishing a microenvironment conducive to immunothrombosis [37]. Neutrophils are key effector cells in immunothrombus formation, continuously responding to mechanical forces including shear and tensile stress [45]. Whether the Piezo1 channel is overly activated by high shear force or its expression is downregulated due to low shear force, it can induce neutrophils to undergo NETosis through different signaling pathways, thereby participating in the formation of immune thrombosis. For instance, high shear stress in stenotic regions activates Piezo1 on the neutrophil surface, enhancing neutrophil sensitivity to platelet-activating factor and lowering the activation threshold, while triggering dose-dependent Ca^2+^ influx. This is followed by increased calpain activity, leading to actin cytoskeletal assembly, depolymerization, and remodeling, culminating in NET formation [46], which in turn promotes platelet adhesion and aggregation [45]. In contrast to the activation pathway under high shear stress, low shear stress downregulates Piezo1 expression, leading to reduced Ca^2+^ concentration, increased histone deacetylase 2 activity, and elevated reactive oxygen species levels, which activate neutrophils and induce NET formation; this process can be reversed by the Piezo1 agonist Yoda1 [47]. Thus, Piezo1 regulates thrombosis through diametrically opposing signaling directions under high versus low shear stress conditions.

NETs are reticular chromatin structures composed of DNA released by neutrophils [37]. Under physiological conditions, NETs capture and neutralize pathogens during host defense; however, excessive or dysregulated NET formation (NETosis) can exacerbate inflammation, activate coagulation factor XII of the intrinsic pathway, and ultimately generate thrombin, thereby promoting thrombotic complications [46,48]. NETosis can be induced by mechanical forces including shear stress, tensile strain, and matrix stiffness [46]. Neutrophil elastase, myeloperoxidase, and cathepsin G within NETs cleave thrombomodulin (an anticoagulant molecule) and tissue factor pathway inhibitor [47], while histones on NETs bind to Toll-like receptors on the platelet surface, activating platelets and inducing pseudopod formation, which firmly anchors platelets to NETs and provides a scaffold for platelet and erythrocyte aggregation [49]. This process can be blocked by mechanosensitive channel inhibitors and activated by the Piezo1 agonist Yoda1. Furthermore, ischemia/reperfusion (I/R) injury has also been implicated in the aforementioned NETs-associated thrombus formation via the Piezo1 pathway. Under conditions of blood stasis and vascular wall ischemia, Piezo1 expression and activity are markedly upregulated, which induces neutrophil extracellular trap (NET) formation, further occluding microvessels and impeding perfusion recovery, thereby establishing a vicious thrombotic cycle [50]. Collectively, these observations indicate that shear stress–induced Piezo1 channel opening in neutrophils serves as a critical link between hemodynamic forces and immunothrombosis [51], with reciprocal activation of platelets and neutrophils forming a positive feedback loop that jointly promotes thrombus progression [52]. Notably, differences in vessel wall composition and hemodynamic environment across distinct anatomical sites may result in varying degrees of dependence on mechanical forces and Piezo1 signaling, underscoring the necessity for individualized therapeutic strategies [53].

## 8. Targeted Antithrombotic Intervention of Piezo1

In recent years, targeting the Piezo1 channel has been recognized as a novel antithrombotic therapeutic strategy in multiple publications [25,48,54]. On one hand, conventional antiplatelet and anticoagulant agents target chemical activation pathways—such as TXA_2_ and ADP—which operate in the mid-to-downstream segments of the thrombotic cascade. In contrast, Piezo1 serves as a critical molecular switch linking hemodynamic aberrations to thrombus formation; thus, targeting Piezo1 constitutes an upstream intervention that can intercept pathological thrombotic signaling before downstream cascades are initiated by pathological mechanical stimuli. On the other hand, conventional antithrombotic agents inevitably compromise physiological hemostasis while inhibiting pathological thrombosis. Currently, no clinical evidence has demonstrated that Piezo1-targeted interventions can selectively suppress pathological thrombosis while preserving physiological hemostatic function, and their safety profile and therapeutic window warrant further evaluation.

Previous studies have shown that non-selective Piezo1 inhibitors can prevent arterial thrombosis and reduce ischemic stroke infarct size [24]. Currently, Piezo1-targeted antithrombotic intervention strategies primarily include pharmacological agonists (e.g., Yoda1 and Jedi1/2), channel inhibitors (e.g., GsMTx4, benzbromarone, and GdCl_3_), natural product-derived traditional Chinese medicine components (e.g., Panax notoginseng saponins, jatrorrhizine, tubeimoside I, and salvianolic acid B), as well as Dooku1 and Xuesaitong (a proprietary Chinese medicine preparation). Given the dual functionality of Piezo1—driving thrombosis under pathological mechanical forces while maintaining vascular homeostasis under physiological conditions—therapeutic modulation does not simply amount to wholesale activation or inhibition, which may incur off-target effects. Instead, it necessitates consideration of the functional differences across distinct hemodynamic environments and cell types. The following sections will elaborate on each class of intervention strategies and their respective molecular mechanisms.

### 8.1. Bidirectional Regulation by Piezo1 Agonists

Currently, the main Piezo1 agonists include Yoda1 and Jedi1/2. The latter, as a more water-soluble type of Piezo1 agonist, can mediate Ca^2+^ influx by activating the Piezo1 channel and reduce FXa production, thereby exerting an antithrombotic effect [46]. However, the application of Jedi1/2 in the field of anticoagulation is currently limited to cell experiments or in vitro studies and lacks effective validation in animal thrombosis models. Yoda1, a well-characterized Piezo1 agonist, enhances the sensitivity of Piezo1 to mechanical stimuli and lowers its activation threshold, thereby potentiating the antithrombotic barrier function of the endothelium [29]. Mechanistically, Yoda1 activates Piezo1 to mimic shear stress-induced Ca^2+^ influx, which in turn activates Akt and ERK1/2 signaling pathways in endothelial cells, significantly reducing laser injury-induced endothelial-associated fibrin antibody deposition and decreasing FXa/thrombin generation [40].

The antithrombotic effects of Yoda1 exhibit pronounced shear stress dependence and bidirectional regulatory characteristics. Under static conditions, 0.25 μM Yoda1 suppresses tumor necrosis factor-α-induced prothrombotic phenotypes—including factor Xa generation, tissue factor expression, and NF-κB activation—while promoting the expression of antithrombotic proteins such as thrombomodulin and endothelial protein C receptor. However, under both laminar and non-laminar shear stress conditions, higher concentrations of Yoda1 are required to achieve comparable protective effects, with significant reduction in FXa and TF protein levels mediating TNF-α generation [55]. SiRNA-mediated knockdown of Piezo1 or transcription factors KLF2 or KLF4 reverses the protective effects of Yoda1 [40].

Under arterial shear flow conditions, however, Yoda1 induces substantial Ca^2+^ influx, promotes endothelial YAP nuclear translocation, and enhances agonist-induced platelet aggregation, secretion, and procoagulant activity involving calpain activation, thereby promoting thrombus formation. Piezo1 gene ablation reverses these phenomena [32,54,56,57]. Addition of Yoda1 to whole blood additionally promotes endogenous thrombin generation via erythrocyte Ca^2+^-dependent volume shrinkage, thereby activating platelets, an effect that can be reversed by rivaroxaban or by removal of extracellular Ca^2+^ [37]. This observation suggests that the antithrombotic application of Yoda1 should take into account the mechanical microenvironment at its site of action.

### 8.2. Antithrombotic Mechanisms of Piezo1 Inhibitors

Extensive studies have demonstrated that pharmacological inhibition of Piezo1 channels significantly reduces thrombosis under arterial shear conditions [23]. The principal Piezo1 inhibitors with antithrombotic activity currently include GsMTx4, benzbromarone, and GdCl_3_.

GsMTx4, a spider venom-derived peptide, effectively inhibits Piezo1 signaling without affecting its expression. By inserting into the lipid bilayer and modifying the lipid–channel interface, GsMTx4 impedes Ca^2+^ entry [55], thereby suppressing platelet Ca^2+^ influx, granule release, integrin activation, and P-selectin expression, while inhibiting platelet shape change, reducing pseudopod formation and immature platelet aggregate generation [30,54], and suppressing PS exposure, PS^+^ microparticle release, and thrombin generation [56], ultimately slowing thrombus formation kinetics and density while prolonging vascular occlusion time.

Furthermore, GsMTx4 downregulates PI3K/AKT and apoptosis-related proteins, upregulates the anti-apoptotic protein BCL-2, and reduces lactate dehydrogenase release [54]. Concurrently, it inhibits platelet mitochondrial reactive oxygen species generation, blocks both cytosolic and mitochondrial Ca^2+^ overload, maintains mitochondrial fusion–fission balance, thereby improving mitochondrial function to better support platelet energy metabolism [28], and attenuating platelet apoptosis. These mechanisms have been validated in prothrombotic disease models such as hypertension and diabetes [58,59]. In animal models, GsMTx4 effectively inhibits high shear stress- and hypertension-induced platelet hyperactivation, reduces arterial thrombus volume and cerebral infarct size [28], with no observed bleeding risk, indicating a favorable safety profile. Notably, the antithrombotic action of GsMTx4 is independent of classical P2X1, Orai1, or TRPC6 channels [32], but rather is achieved by functionally inhibiting the Ca^2+^ influx mediated by Piezo1 and its downstream mitochondrial apoptotic pathway.

In addition to GsMTx4, the small-molecule drug benzbromarone—originally developed as a uricosuric agent—has been shown to partially inhibit Piezo1 activity, abrogate Yoda1-induced effects, and significantly suppress thrombin generation. It uncouples the Piezo1–TMEM16F signaling axis, thereby blocking phosphatidylserine (PS) exposure [45], microparticle shedding, and thrombin production. The therapeutic advantage of benzbromarone lies in its capacity to inhibit pathological PS exposure at low concentrations without completely abolishing the physiological functions of Piezo1, thereby conferring both efficacy and safety in antithrombotic therapy.

Gadolinium chloride is a Piezo1 inhibitor whose activity is dependent on the mechanical environment. It suppresses thrombus formation on collagen surfaces under normal arterial shear stress, resulting in a marked reduction in mean thrombus volume [60]; however, it exerts no effects on platelet morphological changes or aggregation under static conditions. Although GdCl_3_ has been widely used experimentally, its significant toxicities—including genotoxicity and cerebral accumulation—have precluded its development as an antithrombotic agent.

### 8.3. Naturally Derived Piezo1 Modulators

Natural products and their active components, characterized by multi-targeting properties and low toxicity, may modulate Piezo1 expression or activity, thereby influencing shear stress sensing and downstream signaling pathways.

Panax notoginseng saponins (PNS), the principal bioactive constituents of the traditional Chinese herb Panax notoginseng, are widely used for promoting blood circulation and removing blood stasis. Oral administration of PNS inhibits platelet adhesion and aggregation [46]. Mechanistically, PNS antagonizes the effects of Yoda1, suppresses high shear stress-induced platelet calcium influx, and effectively modulates Piezo1-associated downstream proteins [61], thereby interfering with von Willebrand factor binding to platelets and integrin αIIbβ3 activation. Consequently, PNS ameliorates pathological high shear-induced platelet aggregation and arterial thrombosis, as evidenced by a significant reduction in carotid artery occlusion time in animal models [62].

PNS are the principal bioactive constituents of commonly used clinical preparations such as Xueshuantong Capsules and Xuesaitong Injection, which are traditionally employed to promote blood circulation and resolve blood stasis. Liu et al. demonstrated in a carrageenan-induced rat tail thrombosis model that low-dose (50 mg·kg^−1^) and high-dose (150 mg·kg^−1^) Xueshuantong for injection (lyophilized powder) improved blood perfusion in both the common carotid artery and tail microcirculation, respectively, while significantly suppressing platelet Piezo1 protein expression [63]. In an in vitro shear-induced platelet aggregation model, Liu et al. further confirmed that clinically relevant concentrations (0.15 g·L^−1^) of Xueshuantong inhibited shear-induced platelet aggregation by blocking Piezo1-mediated Ca^2+^ signaling and its downstream calpain-2/talin1 pathway [61]. Collectively, these preclinical findings suggest that the antithrombotic effect of Xueshuantong may involve targeting the Piezo1 channel; however, this mechanism has not yet been validated in clinical studies.

Ginsenoside Rb1, one of the most abundant saponins in PNS, accounts for approximately 30–40% of the total saponin content. In animal models, platelet-specific Piezo1 knockout abolished the antithrombotic effect of Rb1 [18], establishing Piezo1 as a critical target through which Rb1 exerts its anti-platelet aggregation effects.

Beyond PNS, multiple additional traditional Chinese medicine-derived compounds have been identified as Piezo1-targeting inhibitors. Jatrorrhizine, an isoquinoline alkaloid primarily found in Coptis chinensis and Phellodendron amurense, suppresses Piezo1 activation and blocks Yoda1-induced Ca^2+^ influx. It also attenuates H_2_O_2_-induced vascular endothelial inflammation and neointimal hyperplasia, manifested by marked downregulation of pro-inflammatory cytokines such as IL-1β and IL-6, alongside upregulation of TGF-β expression, thereby indirectly exerting antithrombotic effects. In Piezo1-deficient endothelial cells, the anti-inflammatory effect of jatrorrhizine was markedly attenuated, suggesting that its function is dependent on the presence of Piezo1 signaling [64].

Tubeimoside I (TBMS1), also known as tubeimoside A, is derived from the dried tubers of Bolbostemma paniculatum. Using intracellular Ca^2+^ measurements and isometric tension recordings, Liu et al. suggested that TBMS1 may act as a Piezo1 channel inhibitor in a Yoda1-dependent manner. Functionally, TBMS1 inhibits Yoda1-induced relaxation of mouse thoracic aortic rings and blocks Piezo1-mediated Ca^2+^ influx and associated physiological functions, thereby exerting antithrombotic effects at both cellular and vascular level [65].

Salvianolic acid B suppresses Piezo1 channel activity, effectively reducing inflammatory cytokine expression and Yoda1-induced foam cell formation, mechanisms that may involve Piezo1-mediated regulation of the MAPK/YAP axis [66]. Additionally, salvianolic acid B inhibits vascular smooth muscle cell proliferation [46], suppresses Yoda1-induced calcium influx, and modulates shear stress-directed endothelial cell alignment, thereby improving endothelial function and maintaining normal flow conditions, which collectively help mitigate the thrombotic risk associated with hemodynamic abnormalities [66].

### 8.4. Additional Intervention Strategies Targeting the Piezo1 Pathway

In addition to the aforementioned modulators, several other agents that target Piezo1 or its downstream signaling cascades have demonstrated antithrombotic potential. Dooku1, an analog of Yoda1, inhibits Yoda1-induced Ca^2+^ influx in HEK293 cells and human umbilical vein endothelial cells, and has been shown to prevent thrombosis associated with sickle cell disease [47,60,67]. The PI3K inhibitor LY294002, through blockade of the PI3K/Akt signaling pathway downstream of Piezo1-Ca^2+^ signals, suppresses hypertension-enhanced platelet aggregation, integrin activation, and P-selectin expression, while concurrently alleviating mitochondrial membrane potential depolarization and reducing lactate dehydrogenase release, indicating that targeted inhibition of the PI3K/Akt axis may globally suppress platelet activation [23]. However, in animal studies, LY294002 has exhibited dose-dependent respiratory depression, somnolence, and reversible dermatitis, along with an extremely short half-life and poor water solubility, which severely limit its in vivo applicability. In the cardiovascular system, this agent can induce early afterdepolarizations in cardiomyocytes and impair myocardial development; in the reproductive system, high doses may affect spermatogenesis, although no embryotoxicity has been observed at lower concentrations. Despite being a key tool compound for studying the PI3K/Akt pathway, LY294002 exhibits significant toxicity and pharmacokinetic limitations. The development of more selective PI3K inhibitors may therefore represent a promising direction for future translational applications. The target pathways and action mechanisms of the aforementioned drugs and components are all exactly as shown in Table 1.

### 8.5. Current Status and Clinical Translation Prospects of Piezo1 Detection Methods

Currently, methods for the direct detection of Piezo1 from patient blood remain at the stage of basic research, with no commercially available kits or standardized protocols. Current detection strategies can be broadly categorized into two types: those assessing the functional activity of the Piezo1 pathway and those measuring Piezo1 protein expression.

In terms of functional activity assessment, Wandi Zhu et al. [69] isolated platelets, erythrocytes, and neutrophils from the peripheral blood of patients with type 2 diabetes mellitus (T2DM). Following stimulation with Piezo1 agonists, they measured the intensity of intracellular calcium influx and found that the calcium response amplitude in patient-derived blood cells was significantly higher than that in healthy controls, suggesting that Piezo1 channels are functionally activated across multiple blood cell lineages. Lennart Kuck et al. [70] employed fluorescent probe technology to measure calcium influx in freshly isolated erythrocytes upon insulin stimulation, thereby indirectly assessing Piezo1 functional status. These methods require equipment such as fluorescence microscopes or flow cytometers and have stringent requirements for sample freshness; their reliability as independent clinical screening indicators remains to be further validated.

With regard to protein expression detection, Suman Guntupalli et al. [71] used FITC-conjugated anti-Piezo1 antibodies to stain peripheral blood platelets and quantified Piezo1 expression levels on the platelet surface via flow cytometry. This approach requires minimal sample volume and offers rapid detection, making it the currently most practical method with clinical application potential. However, it still depends on flow cytometry platforms and lacks commercially available detection kits and standardized operating procedures.

Future efforts should focus on the development of highly specific and high-affinity anti-Piezo1 antibodies for detection purposes, the systematic evaluation of the sensitivity and specificity of these detection methods in predicting thrombotic events, and the clarification of the appropriate patient populations and diagnostic thresholds for clinical application.

## 9. Perspectives

Piezo1 is a critical mechanosensitive ion channel that links mechanical stress to thrombus formation, and targeting this channel holds promise as a novel antithrombotic strategy. However, the translation from basic research to clinical application faces substantial challenges.

Collectively, these limitations suggest that future research should focus on the following directions. First, develop highly selective Piezo1 small-molecule modulators and validate their target engagement using cell-type-specific gene knockout models. Second, currently available tool compounds suffer from limited selectivity and safety concerns. The effects of different agonists and inhibitors vary considerably across cell types, and systematic in vivo toxicity evaluations are lacking. Moreover, some agents are associated with well-defined organ toxicities, which restrict their translational applicability to antithrombotic indications. Third, evaluate the antithrombotic efficacy and bleeding risk of highly selective Piezo1 inhibitors both as monotherapy and in combination with conventional anticoagulants in animal models, to identify rational combination regimens. Fourth, conduct intervention studies in chronic disease animal models of hypertension and diabetes mellitus to obtain dynamic data on Piezo1 regulation under pathological conditions, and analyze the association between Piezo1 expression in blood cells and disease severity as well as clinical outcomes, thereby providing a foundation for clinical translation.

## Figures and Tables

**Table 1:** Antithrombotic agents and active components targeting the Piezo1 pathway.

Category	Drug/ Component	Target/Pathway	Source Type	Mechanism of Action	Category
Agonists	Yoda1	Direct Piezo1 agonist	Synthetic small molecule	Sensitizes Piezo1, mimics shear-induced Ca^2+^ influx in endothelium, activates Akt/ERK1/2 [68], reduces FXa/thrombin generation [40]	Antithrombotic under laminar flow (requires higher concentrations); prothrombotic under non-laminar/arterial shear conditions
	Jedi1/Jedi2	Direct Piezo1 agonist	Synthetic small molecule (improved water solubility)	Activates Piezo1 → Ca^2+^ influx, reduces FXa generation [19,40]	Superior water solubility compared to Yoda1 [24,40]
Inhibitors	GsMTx4	Non-specific/functional inhibition of Piezo1	Spider toxin-derived peptide	Inserts into lipid bilayer to block Ca^2+^ influx; downregulates PI3K/AKT, upregulates BCL-2 [32]; inhibits mitochondrial ROS/Ca^2+^ overload [23]	Effective antithrombosis with no bleeding risk in animal models; human safety requires confirmation [28]
	Benzbromarone	Functional inhibition of Piezo1	Uricosuric agent (small molecule)	Uncouples Piezo1–TMEM16F axis, blocks PS exposure, microvesicle release, and thrombin generation [45]	Inhibits PS exposure at low concentrations, exhibiting a therapeutic window
	GdCl_3_	Non-specific inhibition of Piezo1	Inorganic salt	Inhibits thrombus formation under normal arterial shear stress [60]	Ineffective under static conditions; exhibits genotoxicity and cerebral accumulation, not used clinically
Natural Product Monomers/Extracts	PNS	Functional dependence on Piezo1	Extract from Panax notoginseng	Antagonizes Yoda1 [61], inhibits high shear-induced Ca^2+^ influx; suppresses VWF binding and integrin activation [62]	Orally effective; clinically used formulations (Xueshuantong, Xuesaitong) are safe; low doses suppress Piezo1 expression
	Ginsenoside Rb1	Functional dependence on Piezo1	Major component of PNS	Directly binds Piezo1, inhibits high shear-induced platelet aggregation	Effect abolished in platelet-specific Piezo1-knockout models [31]; no increased bleeding risk observed
	Jatrorrhizine	Functional dependence on Piezo1	Coptis chinensis, Phellodendron amurense	Blocks Yoda1-induced Ca^2+^ influx; attenuates endothelial inflammation (IL-1β/IL-6 decrease, TGF-β increase) [64]	Anti-inflammatory effects diminished in Piezo1-deficient endothelial cells
	Tubeimoside I (TBMS1)	Functional inhibition of Piezo1	Tubers of Bolbostemma paniculatum	Potently inhibits Yoda1-dependent Piezo1 opening, blocks Ca^2+^ influx, inhibits aortic relaxation [65]	Exerts antithrombotic effects at cellular and vascular levels
	Salvianolic acid B	Functional inhibition of Piezo1	Salvia miltiorrhiza	Inhibits Piezo1 → reduces inflammatory cytokines and foam cell formation (MAPK/YAP axis) [66]; inhibits vascular smooth muscle proliferation [49]. improves endothelial alignment	—
Others	Dooku1	Blocks Yoda1 effect (does not directly inhibit Piezo1)	Yoda1 analogue	Inhibits Yoda1-induced Ca^2+^ influx [60,67]	Prevents thrombosis associated with sickle cell disease
	LY294002	PI3K/Akt (downstream)	PI3K inhibitor (tool compound)	Inhibits downstream PI3K/Akt signaling, suppresses platelet activation [23]	Significant toxicity (respiratory depression, dermatitis, cardiac/reproductive toxicity), short half-life

## Data Availability

No new data were created or analyzed in this study. Data sharing is not applicable to this article.
